# Extracellular Vesicles from Healthy Cells Improves Cell Function and Stemness in Premature Senescent Stem Cells by miR-302b and HIF-1α Activation

**DOI:** 10.3390/biom10060957

**Published:** 2020-06-25

**Authors:** Cristina Mas-Bargues, Jorge Sanz-Ros, Aurora Román-Domínguez, Lucia Gimeno-Mallench, Marta Inglés, José Viña, Consuelo Borrás

**Affiliations:** 1 Freshage Research Group, Department of Physiology, Faculty of Medicine, University of Valencia, CIBERFES-ISCIII, INCLIVA, 46010 Valencia, Spain; cristina.mas@uv.es (C.M.-B.); jorge.m.sanz@uv.es (J.S.-R.); aurora.roman@ext.uv.es (A.R.-D.); Lucia.Gimeno@uv.es (L.G.-M.); jose.vina@uv.es (J.V.); 2 Freshage Research Group, Department Physiotherapy, Faculty of Physiotherapy, University of Valencia, CIBERFES-ISCIII, INCLIVA, 46010 Valencia, Spain; marta.ingles@uv.es

**Keywords:** oxygen, redox, physioxia, physiological oxygen concentration, extracellular vesicles, aging, senescence

## Abstract

Aging is accompanied by the accumulation of senescent cells that alter intercellular communication, thereby impairing tissue homeostasis and reducing organ regenerative potential. Recently, the administration of mesenchymal stem cells (MSC)-derived extracellular vesicles has proven to be more effective and less challenging than current stem cell-based therapies. Extracellular vesicles (EVs) contain a cell-specific cargo of proteins, lipids and nucleic acids that are released and taken up by probably all cell types, thereby inducing functional changes via the horizontal transfer of their cargo. Here, we describe the beneficial properties of extracellular vesicles derived from non-senescent MSC, cultured in a low physiological oxygen tension (3%) microenvironment into prematurely senescent MSC, cultured in a hyperoxic ambient (usual oxygen culture conditions, i.e., 21%). We observed that senescent MCS, treated with EVs from non-senescent MCS, showed reduced SA-β-galactosidase activity levels and pluripotency factor (OCT4, SOX2, KLF4 and cMYC, or OSKM) overexpression and increased glycolysis, as well as reduced oxidative phosphorylation (OXPHOS). Moreover, these EVs’ cargo induced the upregulation of miR-302b and HIF-1α levels in the target cells. We propose that miR-302b triggered HIF-1α upregulation, which in turn activated different pathways to delay premature senescence, improve stemness and switch energetic metabolism towards glycolysis. Taken together, we suggest that EVs could be a powerful tool to restore altered intercellular communication and improve stem cell function and stemness, thus delaying stem cell exhaustion in aging.

## 1. Introduction

Cellular senescence is the consequence of several stresses by which cells lose their ability to proliferate [1]. Our group described in recent studies that high oxygen tension (21% O_2_) in primary stem cell culture creates an oxidative stress microenvironment that induces a premature senescence. This senescent phenotype includes morphological alterations, reduced proliferation rate [2], increased p16^INK4a^ mRNA expression and high senescence associated-β-galactosidase activity levels [3]. Furthermore, this oxidative stress-induced premature senescence is also characterized by a minimum expression of SOX2, OCT4, KLF4 and cMYC (OSKM) transcription factors in comparison to physiological oxygen tension levels (3% O_2_), also called physioxia [3]. The accumulation of senescent cells with age contributes to the development of aging pathologies, probably through the secretion of molecules, known as the senescent-associated secretory phenotype (SASP). These molecules have both autocrine and paracrine effects in the neighboring cells, thus compromising tissue microenvironment [4]. Furthermore, it has been reported that the SASP is closely linked to the metabolic state of the cell [4]. As first described by Otto Warburg in 1956, cancerous cells prioritize glycolysis rather than mitochondrial oxidative phosphorylation [5]. Similarly, an altered metabolic state has been associated with senescent cells in culture [6,7].

Extracellular vesicles (EVs), which include exosomes and microvesicles, are released into the extracellular microenvironment by almost all cell types and act as delivery carriers of intercellular communication through the transfer of their contents. Among them, several proteins, lipids, mRNAs, miRNAs and DNA have been described [8] (Vesiclepedia “www.microvesicles.org”). Logically, depending on the characteristics of the cell of origin, EVs may trigger different responses on target cells. These vesicles can transfer signals capable of altering cell fate, cell function and the reprogramming of recipient cells through epigenetic modifications [9].

The induction and maintenance of pluripotency is still challenging because the underlying mechanisms have yet to be fully elucidated. miRs could exert the first strong exogenous impact by changing the transcriptome of a targeted cell [10]. Small noncoding miRs (21–24 nucleotides) are capable of impairing or preventing translation by binding to hundreds of mRNA molecules. The epigenetic status of the genome is regulated by distinct miRs during embryonic growth. Thus, the expression of pluripotency-associated transcription factors seems to be regulated by the epigenetic rebuilding of the genome [11]. Particularly, miR-302 cluster has been reported to be highly expressed in induced pluripotent stem cells (iPSCs) and embryonic stem cells (ESCs), and coordinates proliferation, differentiation, pluripotency maintenance and reprogramming [12]. Accordingly, EVs containing the ESC/pluripotency-specific miRs could be an upcoming strategy for modulating the senescent state, thus eliminating the deleterious effects derived from the well-known hallmarks of aging [13].

We aimed to investigate the beneficial effects of EVs released by physioxia-cultured cells on premature senescent cells. We evaluated senescence biomarkers, mitochondrial bioenergetics, and stemness profile, following microenvironment modulation through EVs.

## 2. Materials and Methods

### 2.1. Dental Pulp Stem Cell Isolation and Culture

Intact third molars were collected from men and women (aged from 15 to 35 years old). All patients were informed and agreed freely to participate and signed the informed consent by contributing the extracted tooth, which was always extracted for reasons independent of this study. The study was approved by the institutional review board of the University of Valencia. Cells cultured from dental pulps did not exhibit any clinical and/or radiological sign or symptom of inflammation and/or infection. To isolate the cells, the pulps were firstly fragmented by trituration, then chemically digested with 2 mg/mL ethylenediaminetetraacetic acid (EDTA) in Krebs–Henseleit buffer, and finally digested with a combination of type I collagenase and type II dispase at a final concentration of 4 mg/mL during 30 min in a humid incubator at 37 °C, 5% CO_2_, and 3% O_2_. The digested pulp fragments were centrifuged at 1000× *g* for 2 min, and the precipitate was resuspended and seeded in culture flasks with complete medium (Dulbecco’s Eagle Modified Medium with low glucose supplement 1 g/L, 10% heat-inactivated fetal bovine serum (FBS) and 1% antibiotic) under the same conditions of temperature and oxygen. Dental pulp stem cell characterization was performed as previously described [2].

After the first passage, the DPSCs were divided in two groups—one group was moved to a humid incubator with an oxygen pressure of 21%, while the other group was kept in the same incubator used for the isolation at 3% oxygen. To perform the experiments, we used hDPSCs at passage 4, referred to as “young”, and hDPSCs at passage 25, referred to as “old”. All of the reagents were purchased from Gibco, Invitrogen, Madrid, Spain.

### 2.2. Extracellular Vesicle Isolation and Characterization

We based our characterization of the EVs population on the definition proposed by the “International Society of Extracellular Vesicles” [14]. hDPSCs were incubated for 48 h with 10 mL complete medium without FBS. After incubation, supernatants were centrifuged first at 300× *g* for 10 min, then at 2000× *g* for 10 min and then at 10,000× *g* for 30 min to remove whole cells, cell debris and aggregates. The supernatant was then ultracentrifuged at 100,000× *g* for 70 min. Pelleted vesicles were suspended in phosphate-buffered saline (PBS), ultracentrifuged again at 100,000× *g* for 70 min for washing, resuspended in PBS and prepared for treatment, transmission electron microscopy and flow cytometry analysis. Acceptor cells were treated with EVs for 48 h (except for miR-302b analysis, where the treatment was interrupted at 12 h or 24 h). In order to quantify the amount of EVs used for treatment, we measured total vesicle protein. On average, an amount of 7 µg of vesicle protein was used for the treatment of 1 million cells.

For transmission electron microscopy (TEM), extracellular vesicles were included in agar and fixed with 2.5% glutaraldehyde, washed with 0.1 M phosphate buffer pH 7.2, and post-fixed with 2% osmium tetroxide in phosphate buffer. After washes with water, they were sequentially dehydrated in 30% EtOH, 50% EtOH, 70% EtOH, 90% EtOH and 100% EtOH. Samples were included in resin and polymerized at 60 °C for 48 h. Ultrathin slides (60 nm) were finally stained with 2% uranil acetate and lead citrate, prior to viewing by TEM, using a Jeol JEM1010 (Croissy-sur-seine, France) microscope at 60 kV. The images were acquired with a digital camera MegaView III with Olympus Image Analysis Software 2.4 (Barcelona, Spain).

For flow cytometry analysis, EV pellets obtained from 10 mL supernatant were resuspended in 100 µL PBS. The EVs were stained with 4 µg/mL of APC-antihuman CD63 (Biolegend, San Diego, CA, USA) for 30 min at 4 °C in darkness. After incubation, positive events were read by fluorescence-activated cell sorting (FACS)–Verse flow cytometry. One unstained sample of the EVs and only one sample with antibody at 4 µg/mL in PBS were used as negative controls. The calibration of the cytometer was assessed using fluorescent particles of standardized size (Nano Fluorescent Particle Size Standard Kit, Spherotech (Lake Forest, IL, USA).

### 2.3. Senescence-Associated β-Galactosidase Activity

Senescence-associated-β-Gal staining was performed using the FluoReporter^®^ LacZ Kit (Molecular Probes, Eugene, OR, USA) following the manufacturer’s instructions. hDPSCs were washed twice with warm PBS and treated with trypsin (Gibco, Invitrogen, Madrid, Spain). Following 1000× *g* centrifugation for 2 min, pelleted cells were resuspended in Staining Medium. Then, 50 µL aliquots of resuspended cells (1 × 10^6^ cells/mL) were placed into an appropriate flow cytometer tube and stained with 50 µL prewarmed fluorescein Di-β-D-Galactopyranoside (FDG) working solution for exactly one minute at 37 °C. FDG loading was stopped by adding 900 µL ice-cold staining medium containing 1.5 µM propidium iodide. The FDG values were read by FACS–Verse flow cytometry until 20,000 events were recorded.

### 2.4. Apoptosis

Apoptosis was assessed using ANNEXIN V FITC Apoptosis detection kit (Immunostep, Salamanca, Spain), following the manufacturer’s instructions. hDPSCs were washed twice with warm PBS and treated with trypsin (Gibco, Invitrogen). Following a 1000× *g* and 2 min centrifugation, pelleted cells were resuspended in 1 × Annexin-Binding Buffer. Then, 100 µL aliquots of resuspended cells (1 × 10^6^ cells/mL) were placed into an appropriate flow cytometer tube and stained with 5 µL Annexin V-FITC and 5 µL propidium iodide for exactly 15 min at 37 °C in darkness. After incubation, 400 µL of 1 × Annexin-Binding Buffer were added. The values were read by FACS–Verse flow cytometry (BDBiosciences, San Diego, CA, USA) until 20,000 events were recorded.

### 2.5. Reactive Oxygen Species

hDPSCs were washed twice with warm PBS and treated with trypsin (Gibco, Invitrogen). Following a 1000× *g* and 2 min centrifugation, pelleted cells were resuspended in serum-free DMEM containing 1 g/L glucose and 1% P/S. Then, 500 µL aliquots of resuspended cells (5 × 10^5^ cells/mL) were placed into an appropriate flow cytometer tube and stained with 5 µL DHR123 (Dihydrorhodamine-123, Thermo Fisher Scientific, Eugene, OR, USA) at a final concentration of 1 µg/mL. The cells were then incubated for 30 min at 37 °C in darkness. After incubation, the intracellular peroxide values were read by FACS–Verse flow cytometry until 20,000 events were recorded.

### 2.6. Mitochondrial Bioenergetics

To assess mitochondria respiration in cells, an XFe96 extracellular flux analyzer (Seahorse Bioscience, Billerica, MA, USA) was used to measure the oxygen consumption rate (OCR) and extracellular acidification rate (ECAR). Then, 7000 h DPSCs were seeded into each well of a 96 XFe Cell Culture Microplate. One day prior to assay, the XFe Sensor Cartridge was hydrated using XFe Calibrant at 37 °C in a non-CO_2_ incubator overnight.

The XFe Cell Mito Stress Test measures key parameters of mitochondrial function by sequential compound injections. One hour prior to assay, the culture medium was changed into unbuffered XFe Base Medium pH 7.4, supplemented with 1 mM pyruvate, 2 mM glutamine and 10 mM glucose, and the culture plate was incubated at 37 °C in a non-CO_2_ incubator for 30 min. On the day of assay, 1 µM oligomycin, 2 µM FCCP, and a 0.5 µM mix of rotenone/antimycin A (final concentrations in each well) were used to measure ATP production, maximal respiration and nonmitochondrial respiration, respectively. Proton leak and spare respiratory capacity were then calculated using these parameters and basal respiration.

The XFe Glyco Stress Test measures glycolytic function in cells by sequential compound injections. One hour prior to assay, the culture medium was changed into unbuffered XFe Base Medium pH 7.4, supplemented with 2 mM glutamine, and the culture plate was incubated at 37 °C in a non-CO_2_ incubator for 30 min. On the day of assay, 10 mM glucose, 1 µM oligomycin and 50 mM 2-Deoxy-D-glucose (DG) (final concentrations in each well) were used to measure glycolysis, glycolytic capacity, glycolytic reserve and non-glycolytic acidification.

After both assays, all measurements were normalized to total protein concentration using Lowry–Folin assay.

### 2.7. mRNA Extraction and RT-qPCR Analysis

Total RNA was isolated from hDPSCs by using TRIzol reagent (Invitrogen, Carlsbad, CA, USA), according to the manufacturer’s instruction. RNA was quantified by measuring the absorbance at 260 nm with NanoDrop 2000. The purity of the RNA preparations was assessed by the 260/280 ratio.

cDNA was synthesized from 2 µg total RNA using the High-Capacity cDNA Reverse Transcription Kit (Applied Biosystems, Foster City, CA, USA). Each reaction was set for 20 µL: 10 × RT Buffer (2 µL), 25 × dNTP Mix 100 mM (0.8 µL), 10 × RT Random Primers (2 µL), Reverse Transcriptase (1 µL), RNase Inhibitor (1 µL), Nuclease-Free Water (3.2 µL) and RNA sample (10 µL). Following the manufacturer’s recommendations, incubation was established for 10 min at 25 °C, followed by 120 min at 37 °C, and then at 85 °C for 5 min and finally cooled to 4 °C to collect the cDNA on a T100 Thermal Cycler (BioRad, Madrid, Spain).

PCR was performed using the Maxima SYBR Green Master Mix kit (Applied Biosystems, Foster City, CA, USA). Each reaction was set for 10 µL: 2 × Maxima SYBR Green/ROX qPCR Master Mix (5 µL), Forward Primer (0.3 µL), Reverse Primer (0.3 µL), Nuclease-Free Water (3.4 µL) and Product from RT reaction (1 µL). Target and control were run in separate wells following the procedure: 10 min at 95 °C and then 40 cycles of denaturation at 95 °C for 15 s and annealing and extension at 60 °C for 1 min per cycle using the detection system 7900HT Fast Real-Time PCR System (Applied Biosystems, Foster City, CA, USA). All the experiments were repeated at least three times for each sample.

SOX2 (Biorad, Madrid, Spain), OCT4 (3′-GATCCTCGGACCTGGCTAAG-5′ and 5′-GACTCCTGCTTCACCCTCAG-3), KLF4 (3′-CCCACATGAAGCGACTTCCC-5′ and 5′-CAGGTCCAGGAGATCGTTGAA-3′) and cMYC (3′-CGCCCTCCTACGTTGCGGTC-5′ and 5′-CGTCGTCCGGGTCGCAGATG-3′) were normalized against the GAPDH (3′-TGAACGGGAAGCTCACTGG-5′ and 5′-TCCACCA- CCCTGTTGCTGTA-3′) housekeeping gene. Relative expression was analyzed using the standard curve method.

### 2.8. miRNA Extraction and RT-qPCR Analysis

Total RNA was isolated from hDPSCs by using TRIzol reagent (Invitrogen, Carlsbad, CA, USA), according to the manufacturer’s instruction. RNA was quantified by measuring the absorbance at 260 nm with NanoDrop 2000. The purity of the RNA preparations was assessed by the 260/280 ratio.

cDNA was synthesized from 2 µg total RNA using the TaqMan MicroRNA Reverse Transcription Kit (Applied Biosystems, Foster City, CA, USA). Each reaction was set for 20 µL: 100 mM dNTPs (0.4 µL), MultiScribe RT (4 µL), 10 × RT Buffer (2 µL), RNase Inhibitor (0.25 µL), Nuclease-free water (3.35 µL), 5 × Pool RT Primers (8 µL) and RNA sample (2 µL). Following the manufacturer’s recommendations, incubation was established for 30 min at 16 °C, followed by 30 min at 42 °C, and then at 85 °C for 5 min and finally cooled to 4 °C to collect the cDNA on a T100 Thermal Cycler (BioRad, Madrid, Spain).

PCR was performed using the TaqMan Universal Master Mix kit (Applied Biosystems, Foster City, CA, USA). Each reaction was set for 10 µL: 20 × TaqMan Small RNA Assay (0.5 µL), 2 × TaqMan Universal PCR Master Mix II no UNG (5 µL), Nuclease-free water (3.5 µL) and Product from RT reaction (1 µL). Target and control were run in separate wells following the procedure: 2 min at 50 °C, 10 min at 95 °C and then 45 cycles of denaturation at 95 °C for 15 s and annealing and extension at 60 °C for 1 min per cycle using the detection system 7900HT Fast Real-Time PCR System (Applied Biosystems, Foster City, CA, USA). All the experiments were repeated at least three times for each sample.

hsa-miR-302b (TaqMan™ MicroRNA Assay) was normalized against the RNU66 (TaqMan™ MicroRNA Assay, Madrid, Spain) housekeeping gene. Relative expression was analyzed using the ∆∆CT method.

### 2.9. Specific Protein Detection by Western Blotting

Total protein was harvested by lysing the cells in a lysis buffer containing a protease inhibitor cocktail (Roche Products, Saint Louis, MO, USA). Protein content was determined by a modified Lowry method [15]. In total, 30 µg of protein from each sample was separated on sodium dodecyl sulfate (SDS)-12.5% polyacrylamide gels and transferred onto a polyvinyldene fluoride (PVDF) membrane (BioRad, Madrid, Spain). Membranes were blocked with 5% bovine serum albumin (BSA) in tris buffered saline (TBS)-0.05% Tween 20 (TBS-T) and incubated with the following antibodies: anti-HIF-1α (1:1000), anti-Tubulin (1:1000) and anti-Mouse (1:10,000). The protein bands were detected by chemiluminescence and analyzed using ImageQuant LAS4000 system (Uppsala, Sweden).

### 2.10. Statistical Analysis

Quantitative variables are expressed as means and SD. Qualitative data are expressed as total number and percentage. Statistical analysis consisted of Student’s T-test for two means. If *n* was not the same in all the groups, the comparison of Scheffé was used. All values are means ± SD of measurements in at least three different cultures (three replicates each). Significance was defined as * *p* < 0.05, ** *p* < 0.01, and *** *p* < 0.001.

## 3. Results

### 3.1. hDPSCs Release Extracellular Vesicles (EVs) to the Medium

**Figure 1:**
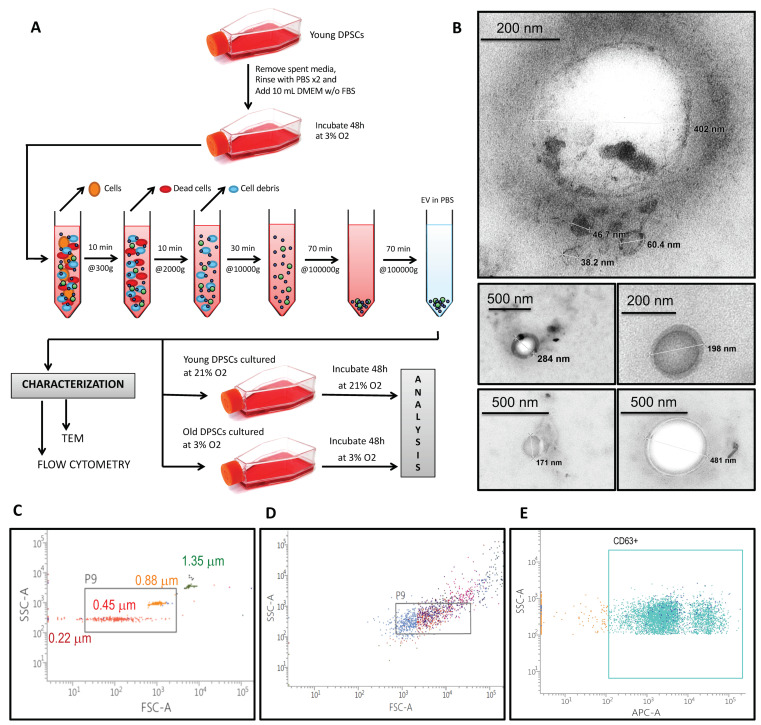
EV characterization. (**A**) Schematic presentation of the experimental workflow to obtain extracellular vesicles. (**B**) Transmission electron microscopy images of EVs derived from hDPSC cultures. Sphere-shaped structures, with 35–500 nm size were identified as EVs. (**C**–**E**) Flow cytometry characterization: (**C**) Standard size beads: 0.22 µm, 0.45 µm, 0.88 µm and 1.35 µm, (**D**) EVs and (**E**) EVs with positive CD63 staining.

Human dental pulp stem cells (hDPSCs) shed a diverse population of extracellular vesicles (EVs). To obtain them, we collected supernatants from hDPSCs after 48 h of culture and we performed differential ultracentrifugation, as shown in Figure 1A. To characterize these EVs, we first performed transmission electron microscopy. EVs can be classified into different groups according to their subcellular origin, physical characteristics (such as size or density), biochemical composition or descriptions of condition or cell of origin [14]. In our case, size measurement showed that the most numerous were small EVs (<200 nm), as shown in Figure 1B [16]. Further characterization by fluorescence activated cell sorting (FACS), using standard size beads of 0.22 µm, 0.45 µm, 0.88 µm and 1.35 µm, revealed that these EVs mainly ranged between 0.2 µm and 1 µm, as shown in Figure 1C. However, beads are perfectly rounded spheres and labelling EVs with fluorescent antibodies might alter their morphology as well as the appearance of aggregates with larger sizes, as seen in Figure 1D. Gating the right population of events revealed that EVs expressed the exosomal marker CD63, as shown in Figure 1E.

### 3.2. EVs Can Modulate Premature Senescence, but Not Replicative Senescence

**Figure 2:**
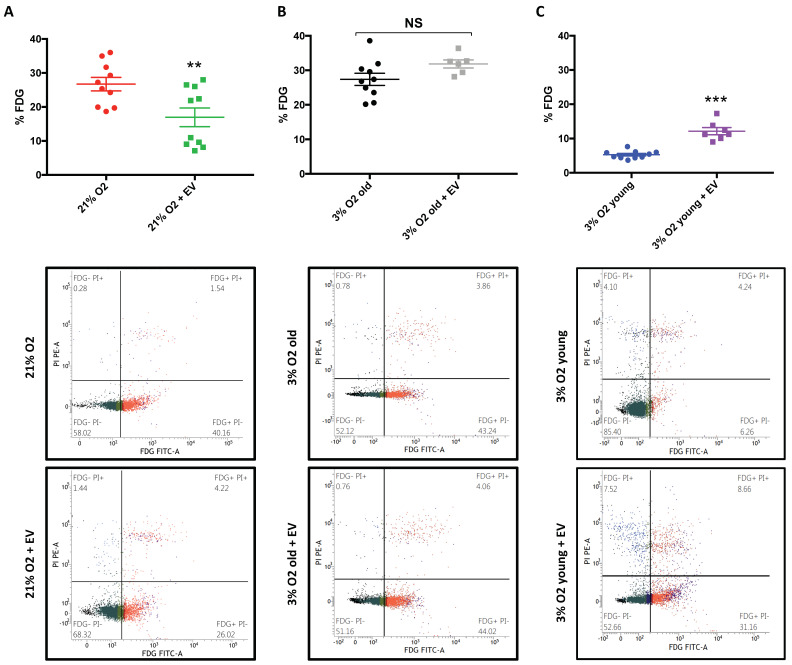
EVs can modulate premature senescence at 21% oxygen. SA-β-galactosidase activity in (**A**) young hDPSCs at passage 5, cultured at 21% O_2_ with or without 48 h incubation with EVs derived from young hDPSCs at passage 5, cultured at 3% O_2_ (*n*_CTL_ = 10 and *n*_EV_ = 10), (**B**) old hDPSC at passage 25, cultured at 3% O_2_ with or without 48 h incubation with EVs derived from young hDPSCs at passage 5, cultured at 3% O_2_ (*n*_CTL_ = 10 and *n*_EV_ = 6) and (**C**) young hDPSCs at passage 5, cultured at 3% O_2_ with or without 48 h incubation with EVs derived from young hDPSCs at passage 5, cultured at 21% O_2_ (*n*_CTL_ = 10 and *n*_EV_ = 6). The data are shown as means ± SD. Statistical significance is expressed as ** *p* < 0.01 and *** *p* < 0.001. NS means no significance.

In previous studies, we demonstrated that the long term culture of hDPSCs under standard culture conditions (21% O_2_) impairs cell proliferation, leading to premature senescence. However, during long term culture under physiological oxygen levels (3% O_2_), hDPSCs only suffer replicative senescence. To determine whether EVs could modify senescence, we analyzed both replicative and premature senescence. We first isolated EVs from young hDPSCs (passage 5) cultured at 3% O_2_. We used sEVs from these cells to treat premature senescent cells (young hDPSCs at passage 5 cultured at 21% O_2_). As shown in Figure 2A, EV treatment significantly reduced SA-β-galactosidase activity compared to those cells that did not receive the treatment. We also used these EVs to treat replicative senescent cells (old hDPSCs at passage 25, cultured at 3% O_2_). As we can observe in Figure 2B, SA-β-galactosidase activity levels were similar in both conditions, suggesting that EV treatment had no beneficial effect on replicative senescence.

Senescent cells assume a specific phenotype, the so-called senescence-associated secretory phenotype (SASP), which is developed after genotoxic stress in culture. SASP is characterized by the secretion of myriad factors, as well as the release of small extracellular vesicles, including exosomes and microvesicles [17,18]. As cell-secreted vesicles have emerged as a mechanism of the intercellular exchange of information and micro ambient modulators, we assessed whether SASP-vesicles could induce senescence. To test our idea, we isolated EVs from the media of young hDPSCs, cultured at 21% O_2_, to treat their counter partners, cultured at 3% O_2_. Figure 2C shows that SASP-vesicles increased SA-β-galactosidase activity levels in young hDPSCs, cultured at 3% O_2_. Therefore, EVs can modulate premature senescence and, depending on their content and/or origin, they can either induce or delay senescence in young hDPSCs.

### 3.3. EV Treatment Switch Mitochondrial Bioenergetic Profile from an Oxidative to a More Glycolytic Metabolism

**Figure 3:**
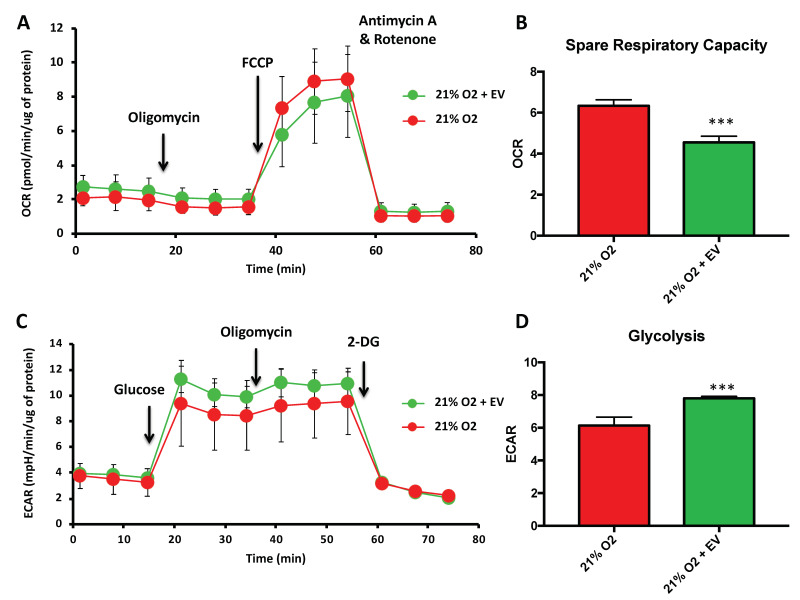
EV reduce mitochondrial oxidative respiration and increase mitochondrial glycolytic activity in premature senescent cells. (**A**) Normalized OCR and (**B**) OCR rates for spare respiratory capacity; (**C**) normalized ECAR and (**D**) ECAR rates for glycolysis in hDPSCs with or without 48 h incubation with EVs derived from young hDPSCs at passage 5, cultured at 3% O_2_. The data are shown as means ± SD (*n* = 45). Statistical significance is expressed as *** *p* < 0.001.

An additional feature of the senescent phenotype is a high metabolic rate [19], and EV treatment could be an important strategy for modulating this metabolic state. We used the Seahorse extracellular energy flux analyzer to determine mitochondrial bioenergetic parameters in hDPSCs. A well-known parameter is the spare respiratory capacity (SRC), which is the difference between the maximum mitochondrial oxygen consumption rate (OCR), measured after FCCP addition, and the basal mitochondrial OCR. Previously, it has been reported that iPSCs and ESCs display reduced mitochondrial SRC, while somatic cells have a significantly higher SRC level [20]. Premature senescent hDPSCs showed lower levels of basal OCR and higher levels of maximum OCR than those treated with EVs derived from physioxia-cultured cells, as shown in Figure 3A. Therefore, EVs reduced the SRC, as shown in Figure 3B, which means that these cells display a low oxidative mitochondrial metabolism after treatment, restoring, in part its stemness potential.

In contrast to somatic cells, pluripotent stem cells show higher rates of glycolysis and lower levels of mitochondrial metabolism, marked by a reduced mitochondrial content [21]. Thus, it is not surprising that, in order to escape senescence, hDPSCs increase their glycolytic metabolism to support rapid cell proliferation, characteristic of young and healthy mesenchymal stem cells. To confirm our hypothesis, we determined the key parameters of the glycolytic function. Glycolysis can be measured as the extracellular acidification rate (ECAR, see methods) reached after the addition of saturating amounts of glucose. The glycolytic reserve is the difference between the maximum ECAR, measured after oligomycin addition, and glycolysis. Premature senescent hDPSCs showed lower extracellular acidification rates than those treated with EVs derived from physioxia-cultured cells, as shown in Figure 3C. Additionally, glycolysis was increased following treatment with EVs, as shown in Figure 3D. Thus, EV treatment induces high glycolytic rates and low OXPHOS levels, suggesting that EVs helped premature senescent hDPSCs to get back to their active pluripotent bioenergetic profile.

### 3.4. EVs Restore Pluripotency in Premature Senescent Cells

**Figure 4:**
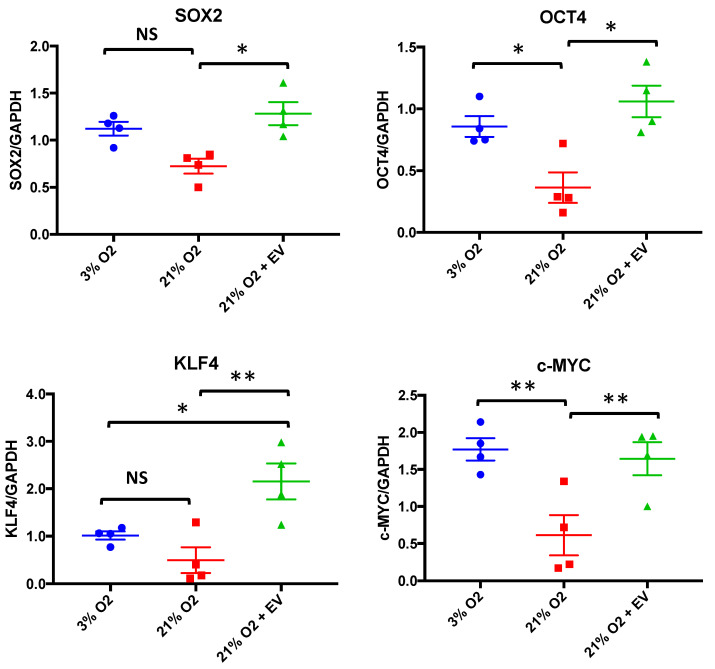
EVs restore pluripotency in premature senescent cells. SOX2, OCT4, KLF4 and cMYC mRNA levels relative expressions in hDPSCs, cultured at 3% O_2_ and hDPSCs, cultured at 21% O_2_ with or without EV treatment. The data are shown as means ± SD (*n* = 4). Statistical significance is expressed as * *p* < 0.05; ** *p* < 0.01. NS means no significance.

The exchange of genetic information through EVs may explain mechanisms involved in the maintenance of stemness or differentiation, as well as in stem cell-mediated tissue repair after injury [9]. According to this, we tested whether EVs derived from physioxia-cultured cells could restore the genetic stemness profile in premature senescent hDPSCs. First, we checked the expression of OSKM (SOX2, OCT4, KLF4 and cMYC) factors [22], which are known to be involved both in the induction and maintenance of pluripotency, respectively. High oxygen tension culture conditions caused a downregulation in the four genes; however, it was only significant for OCT4 and cMYC expression levels compared to physioxia. After EV treatment, we observed a statistically significant increase in all four factors’ expression levels in premature senescent cells, restoring the stemness profile, as shown in Figure 4. Surprisingly, EV treatment increased KLF4 expression levels even more than those of the hDPSCs cultures under physioxia.

### 3.5. Physioxia-Cultured Stem Cell-Derived EVs Modulate OSKM, miR-302b and HIF-1α Expression Pattern

**Figure 5:**
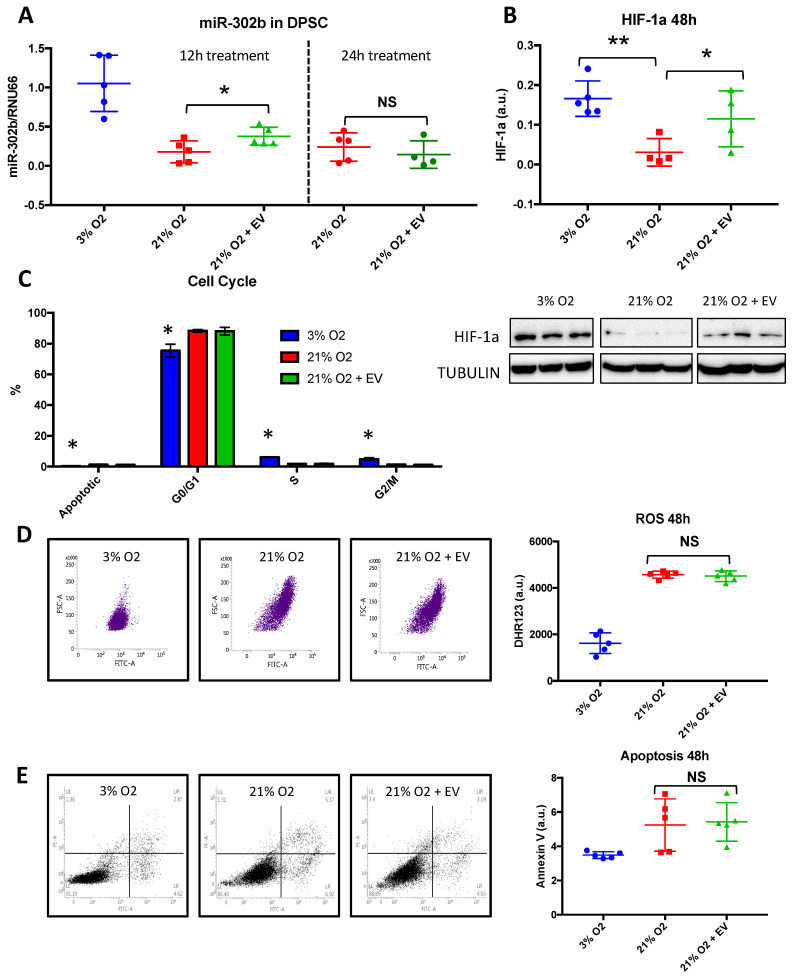
EVs increase miR-302b and HIF-1α expression in premature senescent cells, independently of ROS or apoptosis. (**A**) miR-302b relative expression levels in DPSC at either 12 h or 24 h post-treatment, (**B**) HIF-1α protein levels, (**C**) cell cycle in hDPSCs cultured at 3% O_2_ and hDPSCs, cultured at 21% O_2_ with or without EV treatment. (**D**) ROS levels and (**E**) apoptosis 48 h post-treatment. The data are shown as means ± SD (*n* = 5). Statistical significance is expressed as * *p* < 0.05; ** *p* < 0.01. NS means no significance.

To evaluate whether the impact of physioxia on pluripotency factors is associated with changes in the miRNA pattern, we analyzed the expression of the ESC/pluripotency specific miR-302b. hDPSCs, cultured at physioxia, showed higher expression levels of miR-302b compared to those cultured at 21% O_2_, suggesting that miR-302b expression is influenced by oxygen tension. Furthermore, we detected increased expression levels of miR-302b in hDPSCs cultured at 21% O_2_ after 12 h of treatment with EVs, as shown in Figure 5A. However, these differences were lost after 24 h of treatment, as shown in Figure 5A. After 48 h of treatment with EVs, the hDPSCs cultures also revealed increased levels of hypoxia-inducible factor-1a (HIF-1a), as shown in Figure 5B. It is known that miR-302b possesses pluripotency effects by itself, however, HIF-1α is also able to induce OSKM stem cell markers in adult stem cells [23].

It is also suggested that miR-302b increases cell proliferation and protects cells from oxidant-induced cell death in human mesenchymal stem cells [24]. Therefore, we analyzed ROS levels and cell death in our hDPSCs following EV treatment. As expected, cells cultured at physioxia showed reduced levels of ROS and apoptosis compared to those cells cultured at 21% O_2_. Interestingly, we did not observe any changes in ROS levels, as shown in Figure 5D or apoptosis, as shown in Figure 5E, after the treatment. In parallel with these observations, cell cycle analysis revealed no differences upon EV treatment regarding the G0/G1 phase, S phase and G2/M phase, as shown in Figure 5C. Cells cultured at 3% O_2_ showed reduced levels at the G0/G1 phase, and higher levels at the S and G2/M phases, suggesting an overall increased cell proliferation.

## 4. Discussion

Extracellular vesicle formation allows for the vehiculation of proteins, nucleic acid and lipids, all of them specific to the cell of origin. Therefore, EVs are potentially capable of different biological activities, depending on their content. The transfer of bioactive molecules may change the phenotype and function of the recipient cells. As our results show, introducing EVs derived from a young and healthy micro ambient cell can delay senescence progression. However, not all recipient cells may change phenotypically, as this is the case of replicative senescent cells. These cells have been submitted to serial passaging, thus accumulating damages. On the contrary, our premature senescence model is associated with oxidative stress, which makes young DPSC look older [3]. EV treatment was only useful in premature senescent cells, as no beneficial effect was observed under replicative senescence, suggesting that only premature senescence might be affected. In contrast, SASP-vesicles can induce senescence in young healthy cells, probably through the transfer of molecules involved in senescence pathways and inflammation. In accordance to this, it has been demonstrated that the specific over- or under-expression of certain miRNAs plays a role in senescence through potentially targeting genes on the p53–p21 and p16–pRB pathways [25,26].

As we described in previous studies, 3% O_2_ in vitro condition, or physioxia, maintains the stable expression of OSKM pluripotency factors during serial passages. More specifically, SOX2 and OCT4 are upregulated at early passages and their expression decreases upon passages, suggesting their involvement in stemness induction, whereas cMYC and KLF4 expression at early passages is low and increases with passages, which correlates with their implication in stemness maintenance. On the contrary, 21% O_2_ induces premature senescence, which is accompanied by the rapid downregulation of all of these factors’ expressions in early passages [3]. According to our results, EV treatment caused an overexpression of KLF4 levels, suggesting that EV content not only induces stemness, but also modulates its maintenance. An important role in the induction and maintenance of pluripotency is played by miRs that target mRNAs leading to their cleavage or translational repression. Several ESC-specific miRs have been identified, of which the miR-302 cluster is the most predominant in human ESCs and iPSCs. Specifically, miR-302b has been proven to be overexpressed in different cell types cultured at low oxygen tension, as its promoter is responsive to HIF elements [23]. Additionally, OCT4 and SOX2 are crucial for the transcription of miR-302b in human ESCs [10,27,28].

According to our results, EVs derived from physioxia-cultured cells might be enriched in miR-302b, and its internalization by prematurely senescent cells unleashed the upregulation of OSKM factors. Supporting our findings, it has been proven that miR-302b silences AOF1/2 and DNMT1 activities and, in conjugation with the co-suppression of MECP1/2 and HDAC2, results in global DNA demethylation and chromosomal H3K4me2/3 modifications and subsequently, these epigenetic events induce ESC-specific gene expression, such as SOX2, OCT4 and Nanog [29]. Furthermore, other studies have proven that miR-302b positively regulates OCT4 expression by suppressing nuclear receptor subfamily 2, group F (member 2NR2F2), a member of the nuclear orphan receptor family of transcriptional repressors [30,31].

**Figure 6:**
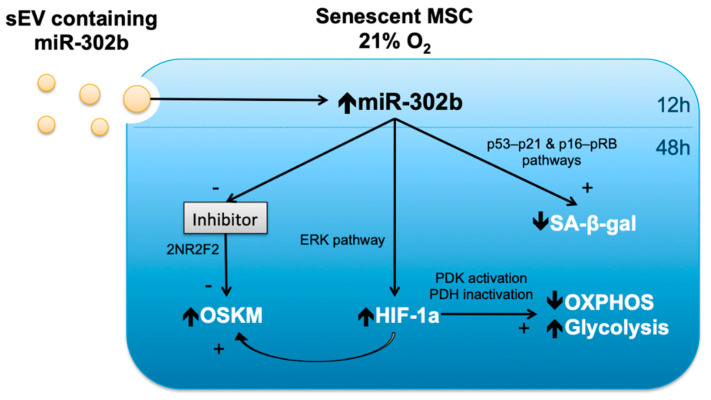
miR-302b downstream effects and mechanisms.

Interestingly, vesicle-encapsulated miR-302b from hDPSCs, cultured under physioxia, also caused an overexpression of HIF-1α in hDPSCs cultured under oxidative stress conditions. In accordance with our results, it has been suggested that miR-302b may induce HIF-1α expression through the ERK pathway [32,33]. Furthermore, HIF-1α induction following this cascade usually takes place 48–72 h after miRNA transfection [34], which matches our findings. As a consequence of HIF-1α activation, reprogramming efficiency is improved by promoting OSKM overexpression for both mouse and human cells [35]. Therefore, the induction of OSKM factors seen in our cells, cultured at 21% O_2_, may be influenced by the EV-encapsulated miR-302b directly, or through HIF-1α modulation, as shown in Figure 6. Besides the direct transfer of EV-encapsulated miR302b to acceptor cells, a positive feedback loop where SOX2 and OCT4 would further stimulate miR-302b endogenous expression has also been suggested [12,36,37]. Lastly, it is possible that EV cargo includes other molecules that could also, in turn, induce endogenous miR-302b expression in DPSC cultured at 21% O_2_. Thus, the exogenous and endogenous upregulation of miR-302b in acceptor cells could be happening at the same time to induce and maintain the stemness properties.

Most stem cells rely on anaerobic glycolysis to maintain their undifferentiated state and for faster ATP generation and the production of cellular building blocks to meet the anabolic demands of high proliferative growth [21]. As they begin to senesce, hDPSCs prioritize a more oxidative metabolism. Supporting this finding, an increase in OXPHOS metabolism has been linked to premature senescence induced by hydrogen peroxide treatment in primary human fibroblasts [38]. Our results show that EVs derived from physioxia-cultured cells are able to switch the metabolism of senescent cells from an oxidative to a more glycolytic one. As mentioned above, these EVs are enriched in miR-302b, which triggers HIF-1α overexpression in hDPSCs cultured at 21% O_2_ upon vesicle internalization. It is known that HIF-1α promotes glycolysis and HIF-1α-dependent pyruvate dehydrogenase kinase (PDK) activation. PDK, in turn, prevents pyruvate oxidation by suppressing the pyruvate dehydrogenase (PDH) complex [39]. Therefore, the metabolic switch seen in our cells cultured at 21% O_2_ could be due to HIF-1α overexpression following miR-302b enriched EV internalization.

Summarizing, microenvironment modulation, through vesicle-encapsulated miR-302b, delays the premature senescence of hDPSCs, and probably reduces SA-β-galactosidase activity by modulating the p53–p21 and p16–pRb signaling pathways. At the same time, miR-302b improves cell stemness by increasing OSKM factor expression, mainly through 2NR2F2 inhibition. Furthermore, miR-302b ameliorates cell function and switches energy metabolism by increasing HIF-1α levels in target cells, possibly through ERK pathway regulation. Figure 6 summarizes the observed downstream effects of miR-302b up-regulation, and the suggested mechanisms involved.

## 5. Conclusions

Taken together, EVs enable micro ambient modulation and cell-to-cell communication via the transfer of functionally relevant biomolecules that regulate senescence, stemness and metabolism. Furthermore, the isolation of EVs derived from young and healthy mesenchymal stem cells is easy, sustainable, reproducible and has a high output, which significantly shortens the time required for cell-based therapies [40]. Thus, they may be useful for cell-free therapeutic purposes.

## Abbreviations

SA	senescence associated
OSKM	OCT4, SOX2, KLF4 and cMYC
OXPHOS	oxidative phosphorylation
EDTA	ethylenediaminetetraacetic acid
FBS	fetal bovine serum
FDG	fluorescein Di-β-D-Galactopyranoside
DG	Deoxy-D-glucose
SDS	sodium dodecyl sulfate
PVDF	polyvinyldene fluoride
BSA	bovine serum albumin
TBS	tris buffered saline
